# ctDNA-Profiling-Based UBL Biological Process Mutation Status as a Predictor of Atezolizumab Response Among *TP53*-Negative NSCLC Patients

**DOI:** 10.3389/fgene.2021.723670

**Published:** 2021-09-07

**Authors:** Jun Lu, Yanwei Zhang, Yuqing Lou, Bo Yan, Benkun Zou, Minjuan Hu, Yanan Wang, Ya Chen, Zhengyu Yang, Huimin Wang, Wei Zhang, Baohui Han

**Affiliations:** ^1^Department of Pulmonary Medicine, Shanghai Chest Hospital, Shanghai Jiao Tong University, Shanghai, China; ^2^Shanghai Institute of Thoracic Oncology, Shanghai Chest Hospital, Shanghai Jiao Tong University, Shanghai, China; ^3^Translational Medical Research Platform for Thoracic Oncology, Shanghai Chest Hospital, Shanghai Jiao Tong University, Shanghai, China; ^4^Clinical Research Center, Shanghai Chest Hospital, Shanghai Jiao Tong University, Shanghai, China

**Keywords:** UBL, biomarker, immune checkpoint inhibitors, atezolizumab, NSCLC

## Abstract

Atezolizumab, an immune checkpoint inhibitor, has been approved for use in clinical practice in non-small cell lung cancer (NSCLC) patients, but potential biomarkers for response stratification still need further screening. In the present study, a total of 399 patients with high-quality ctDNA profiling results were included. The mutation status of ubiquitin-like conjugation (UBL) biological process genes (including *ABL1*, *APC*, *LRP6*, *FUBP1*, *KEAP1*, and *TOP2A*) and clinical information were further integrated. The results suggested that the patients with the clinical characteristics of male or history of smoking had a higher frequency of UBL mutation positivity [UBL (+)]; the patients who were UBL (+) had shorter progression-free survival (PFS) (1.69 vs. 3.22 months, *p* = 0.0007) and overall survival (8.61 vs. 16.10 months, *p* < 0.0001) than those patients with UBL mutation negativity [UBL (–)]; and more promising predictive values were shown in the smoker subgroup and ≤ 3 metastasis subgroup. More interestingly, we found the predictor has more performance in *TP53*-negative cohorts [training in an independent POPLAR and OAK cohorts (*n* = 200), and validation in an independent MSKCC cohort (*n* = 127)]. Overall, this study provides a predictor, UBL biological process gene mutation status, not only for identifying NSCLC patients who may respond to atezolizumab therapy but also for screening out the potential NSCLC responders who received other immune checkpoint inhibitors.

## Introduction

Non-small cell lung cancer (NSCLC) accounts for approximately 85% of all lung cancers (Lu et al., 2019b; Zhang et al., 2020; Lou et al., 2021). Standard therapeutic regimens for first-line therapy have been recommended for NSCLC patients according to the guidelines of the National Comprehensive Cancer Network (Ettinger et al., 2021). Patients harboring EGFR mutations are recommended to receive tyrosine kinase inhibitors (TKIs) such as osimertinib and gefitinib (Zhao et al., 2019; Ettinger et al., 2021); patients harboring ALK mutations are recommended to receive TKIs such as alectinib and crizotinib (Peters et al., 2017; Shaw et al., 2020). However, patients without driver gene mutations usually receive chemotherapy or immunotherapy (Gadgeel et al., 2020; Ettinger et al., 2021). Regarding second-line therapy for NSCLC patients, immunotherapy is potentially suitable for patients with PD-L1 expression (Fehrenbacher et al., 2016; Rittmeyer et al., 2017).

The POPLAR study and OAK studies demonstrated that NSCLC patients who received atezolizumab (one of immune checkpoint inhibitors) as second-line therapy had significantly prolonged overall survival (OS) compared with docetaxel patients, regardless of PD-L1 expression or histology (Fehrenbacher et al., 2016; Rittmeyer et al., 2017). After that, Gandara et al. (2018) found that ctDNA profiling was a potential technology to be used for atezolizumab response stratification in the above NSCLC patients. Furthermore, our previous study suggested that the ctDNA profiling potentially provides more information for immunotherapeutic stratification (Nie et al., 2020). Although an increasing number of studies have introduced that genetic profiling can be used for predicting the efficacy of immunotherapy (Chan et al., 2019; Fabrizio et al., 2021; McGrail et al., 2021), further screening of gene cluster-based biomarkers for immunotherapy is still an urgent problem that needs to be resolved.

Ubiquitin-like conjugation (UBL) biological processes play an important role in cancer development, progression, and therapy. However, the underlying role in immunotherapy is still unclear. In the present study, we found that UBL-enriched gene (*ABL1*, *APC*, *LRP6*, *FUBP1*, *KEAP1*, and *TOP2A*) mutation significantly affected atezolizumab efficacy as second-line therapy in NSCLC patients, and we sought to identify a biomarker for potential use in clinical practice in the future.

## Materials and Methods

### Patients

In the present study, all blood samples were collected from enrolled NSCLC patients from the POPLAR study (NCT01903993) and OAK study (NCT02008227). All clinical trials were performed according to Good Clinical Practice guidelines and those of the Declaration of Helsinki. The purpose of blood sample collection was completely explained to patients or their family members, and the signed informed consent was obtained. In the POPLAR study, 144 of 287 patients received atezolizumab therapy, and 425 of 850 patients in the OAK study received atezolizumab. Collectively, 569 patients were preliminarily selected in the present study.

### ctDNA Profiling

The collected baseline plasma samples from all 569 atezolizumab-treated NSCLC patients underwent uniform procedures for ctDNA mutation calling. The methods for sample collection, storage conditions, cell-free DNA (cfDNA) extraction, library construction, sequencing, analysis, and mutation calling were performed according to a previously published article (Gandara et al., 2018). The clinical information for each patient was downloaded from the online database.^1^

### Screening

**FIGURE 1:**
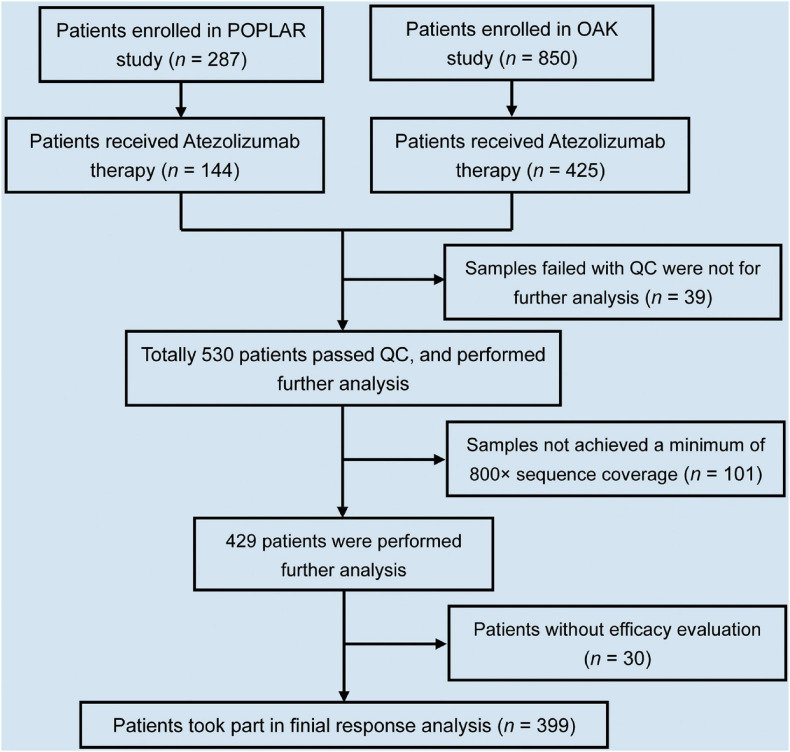
Flowchart of NSCLC patient selection in this study. In total, 1,137 NSCLC patients enrolled in POPLAR and OAK studies. Of 1,137 patients, 569 received atezolizumab therapy. Of 569, 399 patients took part in final analysis after selection.

In total, 569 NSCLC patients had complete clinical information and baseline blood samples. During library construction, 39 of 569 samples failed quality control (including the samples with cfDNA < 20 ng, and the samples failed with templated extension). After sequencing, 101 of 530 ctDNA profiles that did not achieve a minimum of 800× sequence coverage were excluded. Thirty of 429 patients without a definite efficacy evaluation were excluded. Therefore, in this study, 399 patients were selected for final analysis (Figure 1).

### UBL Biological Process

We found 394 cancer-related genes with mutations in all ctDNA samples from 399 patients. Then, the list of these genes was uploaded to the DAVID database for biological process enrichment analysis. UBL biological processes were significantly enriched and mainly included six genes (*ABL1*, *APC*, *LRP6*, *FUBP1*, *KEAP1*, and *TOP2A*).

### Kaplan–Meier Curve Analysis

This method performed as our previous studies (Lu et al., 2019a,c; Chu et al., 2021). The 399 patients who harbored any mutation in UBL biological process genes (*ABL1*, *APC*, *LRP6*, *FUBP1*, *KEAP1*, and *TOP2A*) from ctDNA profiling were defined as UBL (+). A patient who harbored two or more gene mutations of the abovementioned six genes was defined as “UBL (+) harboring 2 or more gene mutations.” If ctDNA profiling detected no mutation in the above UBL biological process genes, the patient was defined as UBL (–). Based on the UBL prediction, the next procedure was stratifying progression-free survival (PFS) and OS by using GraphPad Prism 5 software.

### Subgroup Analysis

According to the different clinical characteristics and driver gene mutation status, we classified the 399 patients into male and female, non-smoker and smoker, non-lung squamous carcinoma (non-LUSC) and LUSC, Asian and White, EGFR (+) and KRAS (+), Eastern Cooperative Oncology Group (ECOG) score = 0 and ECOG score = 1, and metastases ≤ 3 and metastases > 3. Kaplan–Meier curve analysis was performed to calculate the median PFS and median OS and the corresponding log-rank *p*-value. Hazard ratio (HR) was calculated by use of Cox proportional hazards model.

### Validation Analysis

According to the *TP53* mutation status, we classified the 399 patients into *TP53* mutation-positive NSCLC patients and *TP53* mutation-negative NSCLC patients. Similar UBL-based stratification analysis was performed on the NSCLC patients with *TP53* mutation and the NSCLC patients without *TP53* mutation, respectively. For the validation cohort, we selected the *TP53* mutation-negative NSCLC patients who received at least one dose of immunotherapy (atezolizumab, avelumab, durvalumab, ipilimumab, nivolumab, pembrolizumab, or tremelimumab) from the Memorial Sloan Kettering Cancer Center (MSKCC). Different from the POPLAR and OAK cohorts, the mutation information of the NSCLC patients from the MSKCC cohort was derived from tumor tissue DNA (ttDNA). Furthermore, the patients undergo genomic profiling with the Integrated Mutation Profiling of Actionable Cancer Targets (MSK-IMPACT) panel. The patients from the MSKCC cohort just provided the OS information. Therefore, the predictive value of UBL for OS stratification was performed in the validation cohort.

### Statistical Analysis

The Mann–Whitney *U*-test was performed to compare the age difference between the UBL (+) cohort and UBL (–) cohort. The chi-square test was performed to compare the differences of other clinical characters. The log-rank test was used to compare Kaplan–Meier curves during the stratification analysis. The HRs and exact 95% confidence intervals (CIs) are reported. Differences were considered significant at ^∗^*p* < 0.05, ^∗∗^*p* < 0.01, and ^∗∗∗^*p* < 0.001.

## Results

In this study, we identified NSCLC patients harboring UBL biological process gene (*ABL1*, *APC*, *LRP6*, *FUBP1*, *KEAP1*, and *TOP2*) mutations, and this information could be used to stratify patients who might respond to the immune checkpoint inhibitor atezolizumab. Based on our rigorous screening procedure, 399 patients were finally included for screening the responsive biomarker (Figure 1). The analysis of clinical characteristics indicated that there was no association between UBL gene mutation status and age, pathological status, race, driver gene (*EGFR*, *ALK*, and *EML4*) status, metastasis site number, and ECOG score. However, there was a significant increase in UBL gene mutation frequency in the male patients and the patients with smoking history (Table 1). These results suggested that there is a bias in the mutation of UBL biological process genes in NSCLC patients. Whether this phenomenon can be used as a predictor of immunotherapy response is still unclear.

**TABLE 1:** Demographic data of 399 advanced NSCLC patients who received atezolizumab therapy.

**Characteristic**	**UBL (+)**	**UBL (-)**	***p*-value**
**Age (median, range)**	62.5 (41–78)	62.7 (39–82)	0.7894^a^
**Gender (%)**			0.0148^b^
Male	68 (74%)	184 (60%)	
Female	24 (26%)	123 (40%)	
**Smoking (%)**			0.0030^b^
Non-smoker	7 (8%)	65 (21%)	
Smoker	85 (92%)	242 (79%)	
**Pathology (%)**			0.5877^b^
Non-LUSC	68 (74%)	218 (71%)	
LUSC	24 (26%)	89 (29%)	
**Race (%)**			0.1118^b^
Asian	14 (15%)	70 (23%)	
White	70 (76%)	210 (68%)	
Others	8 (9%)	27 (9%)	
**Driver gene status (%)**			
EGFR (+)	4 (4%)	49 (16%)	0.0040^b^
KRAS (+)	7 (8%)	20 (7%)	0.7140^b^
EML4 (+)	0	2 (>1%)	0.4377^b^
**Metastases (%)**			0.1299^b^
≤3	58 (63%)	219 (71%)	
>3	34 (37%)	88 (29%)	
**ECOG (%)**			0.9443^b^
0	32 (35%)	108 (35%)	
1	60 (65%)	199 (65%)	

*^a^Mann–Whitney U-test.*

*^b^Chi-square test.*

**FIGURE 2:**
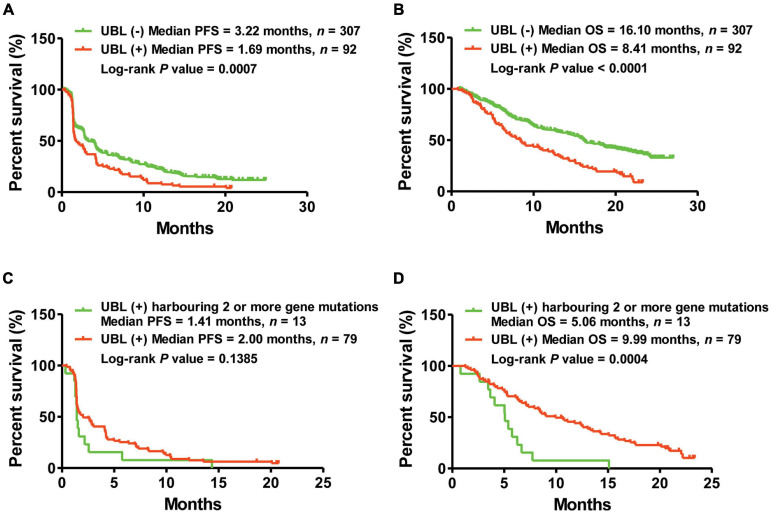
Kaplan–Meier curve analysis of PFS and OS via the predictor of UBL. **(A)** NSCLC patients harboring UBL (+) received median PFS of 1.69 months, while those patients harboring UBL (–) received median PFS of 3.22 months. **(B)** NSCLC patients harboring UBL (+) received median OS of 8.41 months, while those patients harboring UBL (–) received median OS of 16.10 months. **(C)** UBL (+) NSCLC patients harboring two or more gene mutations received median PFS of 1.41 months, while those UBL (+) patients harboring single gene mutation received median PFS of 2.00 months. **(D)** UBL (+) NSCLC patients harboring two or more gene mutations received median OS of 5.06 months, while those UBL (+) patients harboring single gene mutation received median OS of 9.99 months.

Here, we classified 399 atezolizumab-treated NSCLC patients into two cohorts: those who were UBL (+) and those who were UBL (–). Kaplan–Meier curve analysis suggested that the NSCLC patients who were UBL (+) had shorter PFS (UBL (+) vs. UBL (–) = 1.69 vs. 3.22 months, log-rank *p*-value = 0.0007) than the UBL (–) cohort (Figure 2A). Regarding OS analysis, the results showed a more significant difference: the patients in the UBL (–) cohort received more OS benefit from atezolizumab therapy (UBL (–) vs. UBL (+) = 16.10 vs. 8.41 months, log-rank *p*-value < 0.0001) than the patients in the UBL (+) cohort (Figure 2B). More interestingly, in the UBL (+) cohort, the patients who defined as UBL (+) harboring two or more gene mutations received shorter PFS and OS from atezolizumab therapy (PFS: 1.41 vs. 2.00 months, log-rank *p*-value = 0.1385; OS: 5.06 vs. 9.99 months, log-rank *p*-value = 0.0004) (Figures 2C,D). These results indicated that the mutation status of the UBL biological process genes could potentially be used as a predictor of response to atezolizumab as second-line therapy in NSCLC patients.

To further understand the performance of UBL status in subgroups, we first analyzed the sex-induced response difference for NSCLC patients who received atezolizumab. Male NSCLC patients who were UBL (+) had significantly shorter PFS and OS than male patients who were UBL (–), while there was no significant difference between female patients who were UBL (+) and female patients who were UBL (–). UBL (+) patients with a history of smoking had significantly shorter PFS and OS than UBL (–) patients with a history of smoking. For those patients without a smoking history, there was no significant difference between UBL (+) patients and UBL (–) patients. Regarding the non-LUSC subgroup, patients who were UBL (+) had significantly shorter PFS and OS than male patients who were UBL (–) (Table 2).

**TABLE 2:** Subgroup response analysis using the biomarkers of UBL in atezoluzimab-treated patients from OAK and POPLAR cohorts.

	**Median PFS (months)**			**Median OS (months)**		
	**UBL (+)**	**UBL (–)**	***p*-value^*a*^**	**UBL (+)**	**UBL (–)**	***p*-value^*a*^**
Male	1.59	2.86	0.0076	7.89	15.47	<0.0001
Female	2.73	4.01	0.0904	10.04	17.15	0.0314
Non-smoker	1.38	2.79	0.0977	9.99	17.97	0.3255
Smoker	1.74	4.01	0.0009	8.28	15.77	<0.0001
Non-LUSC	1.53	3.19	0.0027	8.28	18.04	<0.0001
LUSC	2.27	3.78	0.0952	8.43	10.05	0.0283
Asian	2.00	2.86	0.7172	20.90	21.26	0.4350
White	1.61	3.61	0.0003	7.29	16.00	<0.0001
EGFR (+)	2.17	2.76	0.6583	16.54	14.23	0.5805
KRAS (+)	1.41	4.07	0.1848	9.00	18.48	0.1633
ECOG = 0	2.78	4.01	0.0691	12.90	22.47	0.0022
ECOG = 1	1.54	2.89	0.0051	6.67	14.88	<0.0001
>3 Metastases	2.27	2.48	0.8028	10.74	10.94	0.2112
≤3 Metastases	1.58	4.17	<0.0001	7.72	18.60	<0.0001

*^a^Log-rank (Mantel–Cox) test.*

**FIGURE 3:**
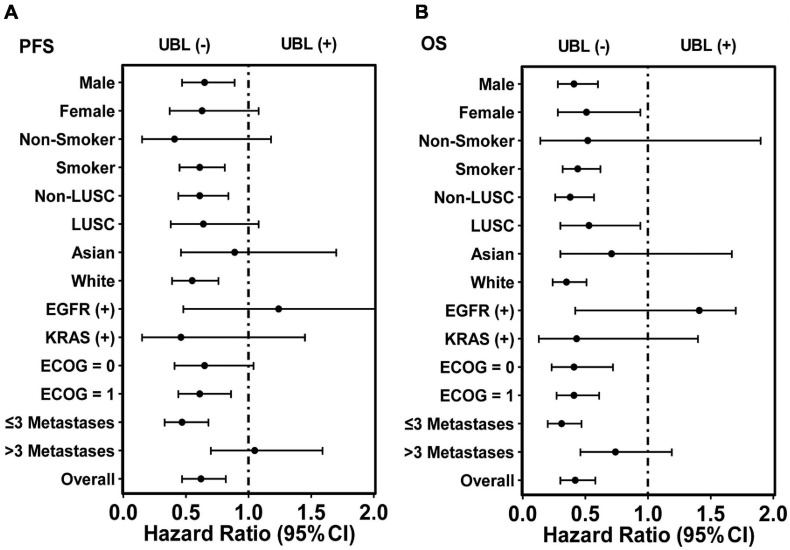
HR analysis of overall 399 patients and corresponding subgroups *via* the predictor UBL-based stratification. **(A)** The predictor UBL potentially decreased HR in nearly all subgroups except the subgroups of EGFR (+) and > 3 metastases when PFS analysis was performed. **(B)** The predictor UBL potentially decreased HR in all subgroups except the subgroup of EGFR (+) when OS analysis was performed.

For LUSC patients, UBL (+) patients had a shorter OS than UBL (–) patients, while there was no difference when comparing the PFS between these two cohorts. Neither UBL (+) nor UBL (–) labeled in Asian patients, and there was no significant PFS or OS outcome difference when patients received atezolizumab therapy. Patients harboring EGFR mutations or KRAS mutations combined with UBL biological process gene mutations who received atezolizumab therapy had PFS and OS outcomes that were similar to those of patients without UBL biological process gene mutations. Regarding the ECOG score = 1 subgroup, patients who were UBL (+) had significantly shorter PFS and OS than those without UBL (–). For the ECOG score = 0 subgroup, UBL (+) patients had a shorter OS than UBL (–) patients, while there was no difference when comparing the PFS between the two cohorts. Interestingly, UBL significantly stratified responders from non-responders in the ≤ 3 metastasis subgroup (Table 2). These results suggested that UBL biological process gene mutation status could potentially be used as a predictor of response to atezolizumab therapy in NSCLC patients, especially in the male, smoker, non-LUSC, White, ECOG score = 1, and ≤ 3 metastasis subgroups. In addition, our analysis focused on HR indicated that the UBL status predictor remarkably distinguished patients with an atezolizumab response and reduced risk of death in the White subgroup and the ≤ 3 metastasis subgroup (Figure 3).

**FIGURE 4:**
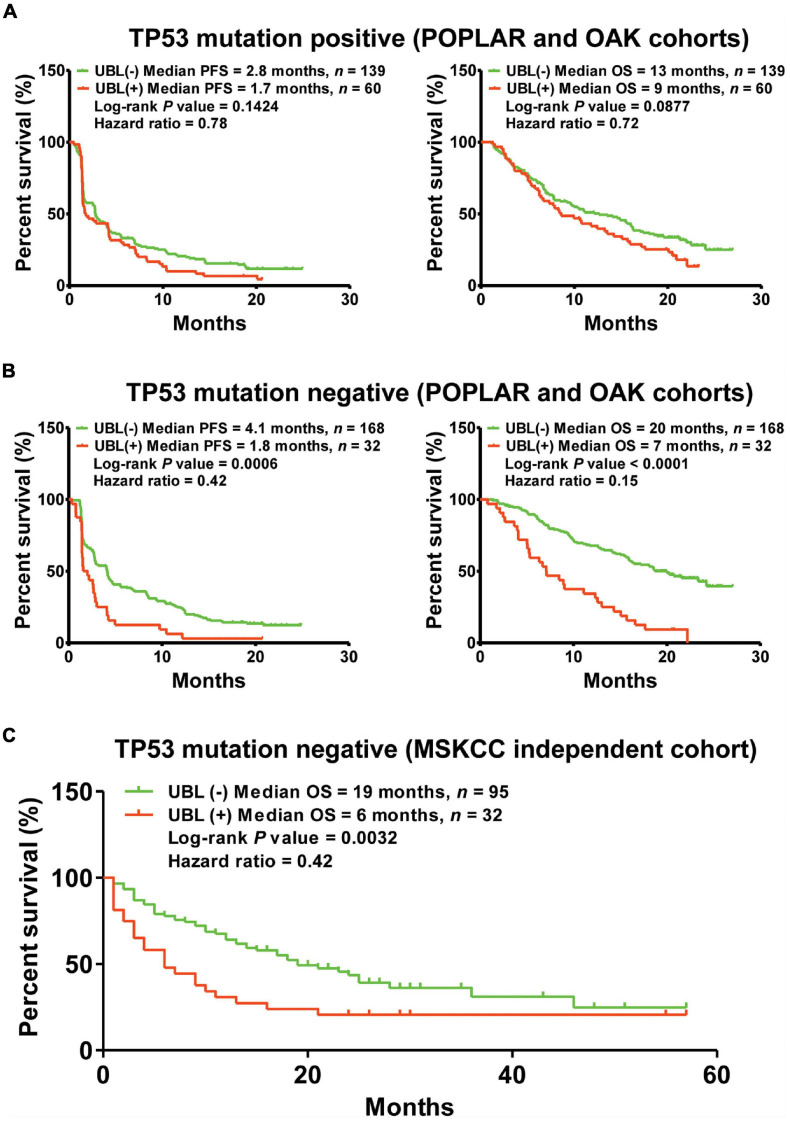
The predictor of UBL for immunotherapeutic stratification in *TP53* mutation-based subgroups. **(A)** The predictive values of UBL in the NSCLC patients harboring *TP53* mutation. **(B)** The predictive values of UBL in the NSCLC patients without harboring *TP53* mutation. **(C)** An independent MSKCC cohort was used to validate the predictive value of UBL in the *TP53*-negative NSCLC patients who received immunotherapy.

The frequency of *TP53* mutation accounts for the highest grade (about 50%) in NSCLC. Whether the predictor of UBL plays a differential role between *TP53-*positive NSCLC and *TP53-*negative NSCLC is still unclear. To further understand the performance of UBL status in *TP53* mutation-based subgroups, here we analyzed the *TP53* mutation-induced response difference for NSCLC patients who received atezolizumab. As shown in Figure 4A, there is no significant distinguishing values of the predictor UBL among the NSCLC patients harboring *TP53* mutation. Interestingly, the predictor UBL showed more predictive values in the *TP53* negative NSCLC patients than that in overall NSCLC patients (Figures 3, 4B). To validate whether the predictor UBL can be validated in an independent cohort, we used the NSCLC patients who received immunotherapy from MSKCC center as the validation cohort. Results demonstrated that the *TP53* mutation-negative NSCLC patients with benefited OS outcome can be screened out significantly *via* the predictor UBL-based stratification (Figure 4C).

## Discussion

Immunotherapy has changed clinical practice in NSCLC (Eguren-Santamaria et al., 2020; Pinheiro et al., 2020; Ettinger et al., 2021). With the clinical application of immune checkpoint inhibitors in NSCLC, an increasing number of clinical problems have surfaced (de Miguel and Calvo, 2020; Haanen et al., 2020; Ramos-Casals et al., 2020; Shankar et al., 2020). Among these emerging clinical problems, how to screen out responders using a predictor is always confusing for clinical physicians and scientists (Darvin et al., 2018; de Miguel and Calvo, 2020). Therefore, in the present study, we sought to identify a ctDNA profiling-based predictor that might be used to stratify responders to the immune checkpoint inhibitor atezolizumab as second-line therapy among NSCLC patients.

Previous studies have demonstrated that genetic profiling can potentially be used as a biomarker for response to immune checkpoint inhibitors (Gandara et al., 2018; Samstein et al., 2019; Alborelli et al., 2020). However, an increasing number of scientists have provided different viewpoints about the usage of genetic profiling for predicting therapeutic efficacy in immunotherapy (Fabrizio et al., 2021; McGrail et al., 2021). With the development of technology, blood-based next-generation sequencing has opened a new field of view for biomarkers predicting response to immune checkpoint inhibitors (Gandara et al., 2018). Our previous study showed that the ctDNA profiling potentially provides more information for immunotherapeutic stratification (Nie et al., 2020). Samstein et al. (2019) and McGrail et al. (2021) demonstrated that genetic profiling plays different roles in different cancer types. Furthermore, the biomarkers including PD-L1 and microsatellite instability also have some questions that need to be resolved (Chang et al., 2018; Gandara et al., 2018). Therefore, predictor discovery for immunotherapy has just started, and there is much unknown information that needs to be explored.

In this study, we provided evidence that UBL could be used as a predictor to screen out responders from non-responders among NSCLC patients who received atezolizumab as second-line therapy. Our results indicated that NSCLC patients who were male and had a smoking history had a higher frequency of being UBL (+). This is a very interesting phenomenon. Although there is not enough evidence to confirm the association of the above characteristics, we still have a reason to speculate that smoking potentially contributes to UBL biological process gene mutations according to previously reported relationships between smoking and genetic variation (Nagahashi et al., 2018). Further analysis demonstrated that NSCLC patients who were UBL (+) had shorter PFS and OS than patients who were UBL (–). Either PFS analysis or OS analysis showed very promising results for UBL biological process gene mutation status to be able to significantly screen out responders from non-responders.

The roles of UBL biological process gene mutation status in screening out responders in subgroups contributed an important composition in the present study. According to our results, 70.4% of patients were White, and 69.4% of patients had ≤ 3 metastases among all 399 NSCLC patients. Some of the bright points in the subgroup analysis are that UBL biological process gene mutation status can significantly distinguish responders and non-responders when used in the above subgroups. However, why this phenomenon occurred still requires further study.

Based on existing evidence, there may be great differences in tumor biology between patients with NSCLC harboring *TP53* mutations and those without *TP53* mutations (Mogi and Kuwano, 2011; Jamal-Hanjani et al., 2017; Bailey et al., 2018; Birkbak and McGranahan, 2020). Interestingly, we found the UBL biological process gene mutation status has a very promising predictive value for screening out responders from non-responders both in the POPLAR and OAK cohorts, as well as the MSKCC cohort.

Collectively, this study provided a predictor, UBL biological process gene mutation status, that could be used to distinguish potential responders from non-responders to atezolizumab as second-line therapy among NSCLC patients, with a more promising predictive value in *TP53* mutation-negative subgroups. Furthermore, the predictor UBL biological process also potentially screen the responders from non-responders for the *TP53* mutation-negative NSCLC patients who received other immune checkpoint inhibitors.

## Data Availability Statement

The datasets presented in this study can be found in online repositories. The names of the repository/repositories and accession number(s) can be found in the article/supplementary material.

## Ethics Statement

The studies involving human participants were reviewed and approved by the Shanghai Chest Hospital. The patients/participants provided their written informed consent to participate in this study.

## Author Contributions

BH, WZ, HW, and JL conceived and designed the experiments. JL, YZ, YL, BY, BZ, MH, YW, YC, ZY, and WZ performed the clinical analysis, bioinformatics analysis, and statistical analysis. JL, YZ, and YL generated the figures and tables. JL wrote the manuscript. BH revised the manuscript. All authors contributed to the article and approved the submitted version.

## Conflict of Interest

The authors declare that the research was conducted in the absence of any commercial or financial relationships that could be construed as a potential conflict of interest.

## Publisher’s Note

All claims expressed in this article are solely those of the authors and do not necessarily represent those of their affiliated organizations, or those of the publisher, the editors and the reviewers. Any product that may be evaluated in this article, or claim that may be made by its manufacturer, is not guaranteed or endorsed by the publisher.

## Footnotes

1
https://clinicalstudydatarequest.com/Study-Sponsors/Study-Sponsors-Roche.aspx
